# Burden of geriatric and cognitive disorders and the impact of integrated care models on morbidity, functional decline, and health service utilization among older adults

**DOI:** 10.3389/fmed.2026.1820760

**Published:** 2026-06-29

**Authors:** Lili Chai, Ning Gou

**Affiliations:** Shaanxi Provincial People’s Hospital, Xi’an, Shaanxi, China

**Keywords:** cognitive impairment, functional decline, geriatric syndromes, hospitalization, integrated care, multimorbidity, retrospective cohort

## Abstract

**Background:**

The health conditions of elderly people who have multiple chronic illnesses and geriatric syndromes lead to severe health problems, which result in decreased ability to function and increased need for medical services.

**Methods:**

The study used a retrospective cohort design with propensity score matching and multivariable modeling to examine the impact of different care models on outcomes over a 24-month period. Five outcomes including hospitalization rates and functional decline rates through ADL and IADL assessments, cognitive decline rates, mortality rates, and service usage rates.

**Results:**

The matching process achieved balanced baseline characteristics between groups because SMD measurements stayed below 0.05. Geriatric syndromes had high prevalence rates with 40.0% cognitive impairment, 32.0% frailty, 32.5% depression, 49.0% polypharmacy, and 68.5% multimorbidity. The health service utilization showed substantial levels, which included 92 hospital admissions per 100 person-years and 134 emergency visits per 100 person-years. 31.4% of participants experienced functional decline in activities of daily living ADL and 42.7% suffered a similar decline in instrumental activities of daily living IADL while EQ-5D showed quality-of-life deterioration of −0.04. Integrated care provided benefits which decreased hospital admissions (IRR 0.78, 95% CI 0.71–0.87) and 30-day readmissions (OR 0.81) and reduced length of stay (−1.2 days). Cognitive decline underwent partial protection (MoCA change +0.86; dementia conversion HR 0.71), and mortality decreased (HR 0.76). Multivariable models show that frailty (OR 2.21) and cognitive impairment (OR 1.42) served as strong predictors of functional decline. The protective effects of integrated care continued to exist (OR 0.81). Mediation analysis suggested that approximately 25% of the observed advantage came from reduced frailty.

**Conclusion:**

Older adults exhibit high burdens of geriatric syndromes and healthcare utilization, which leads to their functional and cognitive decline. Integrated care models reduce hospital admissions and delay cognitive decline while increasing patient survival because they help manage frailty and provide unified treatment.

## 1 Introduction

The global population is aging at an unprecedented rate, which leads to increasing rates of multimorbidity and geriatric syndromes and cognitive disorders that create major difficulties for both individuals and healthcare systems. Older adults show multiple health issues, which include frailty, cognitive impairment, depression, polypharmacy, and functional decline, which together raise their chances of experiencing hospital stays, institutionalization, and death (1). The multiple conditions that people experience simultaneously create a situation where their disabilities increase and their quality of life decreases (2). The older population uses health services at high rates because they require continuous emergency medical treatment, their chronic conditions become worse, and their physical abilities decline (3). The traditional healthcare approach, which emphasizes treating specific diseases, does not meet the complete healthcare requirements of this patient group (4).

The worldwide health burden and medical costs of elderly people and cognitive disorders constitute a significant part of global health and treatment costs (5). Cognitive impairment and dementia lead to progressive decline in independence, which requires increased support from others, whereas frailty and functional decline establish a risk pathway that results in falls, disability, and hospital admissions (6). The clustering of these conditions shows how aging-related health problems need complete treatment approaches that focus on their complex relationships (7). Previous studies have demonstrated that multimorbidity and geriatric syndromes are strong predictors of health service utilization and mortality, which creates a need for medical treatments that will treat both health problems and daily living skills (8).

Integrated care models serve as an effective solution for handling intricate medical requirements of elderly populations through their ability to unite various medical specialties and implement preventive measures while focusing on individual patient needs (9). The purpose of these models is to establish better care continuity while they work to solve health issues that stem from various medical, functional, and psychosocial aspects. Previous research demonstrated that integrated care programs decrease hospital admissions and increase functional improvements and life quality for elderly patients (10). Current research continues to explore the complete extent and operational methods that these advantages create, particularly for people who experience severe geriatric syndromes and cognitive impairments (11). Health policymakers and healthcare organizations need to understand how integrated care affects patient sickness rates and their ability to function and use medical services (12).

The evidence base for integrated care shows substantial gaps because researchers have not studied its long-term health effects on older populations (13). The retrospective and observational studies have delivered useful findings that demonstrate different ways to carry out research and define study results (14). Researchers need to study how geriatric syndromes, cognitive impairment, and health service use interact because this knowledge will help them find effective intervention methods (15, 16). The study examines relationships between integrated care and health outcomes in older adults by utilizing a large retrospective cohort study (17, 18).

The current study examines how geriatric and cognitive disabilities affect healthcare costs and how integrated care systems affect patient health outcomes, their ability to function, and their cognitive abilities, and their use of medical services.

## 2 Methodology

### 2.1 Study design and setting

The researchers conducted a retrospective cohort study that examined data from senior patients who received treatment at a major hospital network during January 2024 to December 2025 observation period. The study aimed to assess how integrated care models impacted various health outcomes, which included changes in morbidity rates, functional abilities, cognitive development, and health service usage. The research team used electronic health records and national mortality registries to extract data, which enabled them to establish complete outcomes and conduct sustained follow-up assessments.

### 2.2 Study population

The study examined a group of 2,300 participants who were 65 years old and above and who showed documented usage of healthcare services throughout the research duration. Participants were divided into two separate groups, which included integrated care (*n* = 1,110) and standard care (*n* = 1,190) groups. The researchers used the propensity score matching method to create two groups with equal baseline characteristics, which resulted in 980 matched participants for each group. The researchers used age, sex, multimorbidity, frailty, cognitive impairment, and polypharmacy as matching variables to decrease selection bias while increasing matching accuracy.

### 2.3 Group allocation and classification of care delivery models

The participants retrospectively fell into two groups depending on the type of care delivery models noted in their electronic health record systems throughout the duration of the study. The participants who followed a standard medical management plan that was mainly dependent on outpatient physician care, specialist referrals, and fragmented hospitalization processes fell under the category of Standard Care Group. The participants who fell under the category of Integrated Care Group belonged to a coordinated multidisciplinary care pathway for the elderly, involving a detailed geriatric assessment, medication reconciliation, cognitive and frailty screening, rehabilitation plans, social support assessments, and long-term follow-ups.

Groups assignment was carried out based on institutional care program files, multidisciplinary clinic attendance, care coordination notes, and healthcare use pathways found from the electronic medical records. Participants enrolled into the integrated care pathway had to meet criteria including documentation of engagement in at least three aspects such as geriatric specialist assessment, coordinated nursing care, pharmacological review, physical or occupational therapy input, cognitive or psychiatric evaluation, nutrition counseling, or structured transitional care planning after hospital discharge.

Patients aged ≥ 65 years with complete demographic, clinical, and follow-up information were selected for the analysis. Exclusion criteria included absence of necessary data, presence of terminal illness, advanced malignancy with life expectancy of < 6 months, or lack of follow-up information. As this study design was nonrandomized, participants assigned to different groups were not randomized, thus, a propensity score matching and multivariate analysis was conducted afterward to control for any baseline differences.

### 2.4 Data sources and variables

The study obtained data from electronic health records, clinical registries, and administrative databases. The researchers assessed baseline variables, which included demographic data, comorbidity information, geriatric syndrome identification, and medication usage. The study assessed health service utilization through hospital admissions, readmissions, and ICU stays, and measured functional status through Activities of Daily Living (ADL) and Instrumental Activities of Daily Living (IADL), and cognitive function through Montreal Cognitive Assessment (MoCA), and tracked mortality rates. The researchers used standardized definitions and validated instruments to achieve consistent measurement results.

### 2.5 Definitions of variables and study findings

#### 2.5.1 Demographic variables

The participant’s age was measured using years at the time of baseline assessment. The sex variable was measured as male or female depending on medical records. Education status was measured using completion of 12 years or more of formal education. Living status was measured as living alone or with others.

#### 2.5.2 Clinical and comorbidity measures

The body mass index (BMI) was calculated as weight in kilograms divided by square of the height in meters (kg/m^2^). Obesity was classified as a BMI ≥ 30 kg/m^2^. Multimorbidity was defined as the existence of two or more chronic conditions, recorded in the electronic medical record. In addition to this definition, comorbidity burden was measured by means of the Charlson Comorbidity Index (CCI).

These measures included but not limited to hypertension, diabetes, cardiovascular disease, chronic kidney disease, chronic obstructive pulmonary disease, and other chronic diseases.

#### 2.5.3 Frailty index

The Frailty Index (FI), a measure of accumulated health deficits, was used for assessing frailty. The individuals with FI ≥ 0.25 were considered frail. An increasing value of FI suggested more pronounced physiological frailty.

#### 2.5.4 Cognitive and psychological measures

For the assessment of cognitive function, the Montreal Cognitive Assessment (MoCA) test was conducted, which is scored on a scale of 0–30 points, where a smaller number indicates poor cognitive performance.

Diagnosis of dementia was made based on the presence of clinical diagnosis noted in the medical history.

Geriatric Depression Scale (GDS) was used to assess depression severity, where GDS ≥ 10 signified depression.

#### 2.5.5 Measures of functional status

For the assessment of ADL, a standard six-point scale rating independence in basic activities of daily living was used. For the assessment of IADL, an eight-point functional scale rating independent living, including shopping, traveling, and managing medications, was used. The presence of functional deterioration was characterized by the loss of at least one point in either ADL or IADL scales.

#### 2.5.6 Geriatric syndromes

The presence of geriatric syndromes was determined by review of electronic medical records, geriatric assessment, and diagnosis based on clinical records at baseline. The syndromes studied included cognitive impairment, dementia, frailty, depression, falls, and polypharmacy. Cognitive impairment was assessed using the Montreal Cognitive Assessment (MoCA), and MoCA scores obtained from patient records are presented on a scale ranging from 0 to 30.

The condition of polypharmacy included the use of at least five prescribed medications simultaneously. The condition of falls was indicated by one or more documented cases of falls occurring during the last year. The severity of chronic pain was measured based on the Visual Analog Scale. Malnutrition was determined based on the Mini Nutritional Assessment–Short Form test. Impairments of vision and hearing were detected based on clinical findings or the use of corresponding assistive devices. Social isolation included the presence of social isolation or solitude.

#### 2.5.7 Quality-of-life measures

Health-related quality of life was measured by using the EuroQol-5 Dimension (EQ-5D) scale. Self-assessed health condition was measured by standardized Likert scale questionnaires; high values indicated low levels of self-perceived health.

#### 2.5.8 Healthcare utilization measures

Data on hospitalizations, ED utilization, office visits, ICU admissions, SNF use, and home health care were collected from healthcare utilization files for 24 months. Length of hospitalization was recorded as number of days per admission. Thirty-day hospitalization re-admission was considered if hospitalization occurred within 30 days of discharge.

#### 2.5.9 Cognitive and mortality outcomes

Cognitive impairment was defined as change of ≥ 2 points in MoCA score over follow-up. Dementia onset was defined as occurrence of new diagnosis of dementia during follow-up. Overall mortality, cardiovascular mortality, and infection-related mortality rates were determined from hospital files and death certificates.

### 2.6 Definition of integrated care

Integrated care was defined as a well-coordinated multi-disciplinary approach to geriatric management in terms of continuous care provision for medical, functional, cognitive, and psychosocial needs. The patients in the integrated care arm were under the guidance of an interdisciplinary team comprising geriatricians, general practitioners, geriatric nurses, pharmacists, physiotherapists, occupational therapists, social workers, and dietitians. The role of the geriatricians was to coordinate comprehensive geriatric assessment (CGA), develop care plans, and manage complicated conditions associated with aging. General practitioners were responsible for the provision of regular primary care services and managing chronic diseases. Geriatric nurses carried out regular assessments, patient education, symptom monitoring, and coordination of care. The role of the pharmacists included conducting structured medication review, identification of inappropriate drugs, and optimal use of pharmacotherapy. Physiotherapists were involved in assessing patients’ mobility, balance, and physical functioning; they implemented exercise and rehabilitation programs customized for each patient. Occupational therapists assessed patients’ activities of daily living and provided interventions to improve functional ability and safety in the home environment.

Study participants were defined as being within the integrated care group based on confirmation from patient medical records that at least three core aspects of the integrated care approach had been used (assessment, follow-up, and medication review). The rest of the participants received conventional care management services.

### 2.7 Assessment of self-rated health status

The self-rated health status was assessed through the use of patient-reported data, which were normally recorded through the course of the routine outpatient clinic visits and also as part of the comprehensive geriatric assessments. Patients whose cognitive function was adequate, determined based on clinical observation and communication ability, completed their self-rated health questionnaires independently. Out of the total number of patients involved in the study, roughly 79.3% of the subjects were capable of completing self-reports of their health condition. On the other hand, in cases where there were severe cognitive impairments, dementia, communication problems, and/or inability to respond to the questionnaire properly, the self-rated health information was sourced from the primary caregiver or an authorized proxy respondent and included in the record as well.

### 2.8 Statistical analysis

Descriptive statistics described the initial attributes of the study population. The study used propensity score matching to reduce confounding factors, which the researchers used to determine the relationship between different care models and their related outcomes through multivariable regression analysis. The research utilized negative binomial models to examine hospital admission, count data, and used logistic regression to study functional decline binary outcomes, while Cox proportional hazards models studied mortality rates. The model diagnostics procedure used dispersion statistics together with the Akaike Information Criterion (AIC) and calibration tests. The research used three methods: complete case analysis, inverse probability treatment weighting, and random effects models to test study results for their reliability. The research used subgroup and mediation analysis methods to investigate study effect differences together with potential causal relationships.

### 2.9 Bias mitigation and data quality

The research team used matching methods together with sensitivity testing because of their effectiveness in reducing bias that arises from retrospective study designs. The team used multiple imputation to handle missing data when it proved suitable for the research needs. The study used validated registry data to determine study outcomes, which helped to decrease the chances of misclassification. The researchers proved analytic validity through tests, which examined proportional hazards assumptions and model calibration tests.

### 2.10 Ethical considerations

The research used de-identified historical data, which complied with both institutional data management regulations and ethical research standards. The study maintained patient confidentiality while conducting research without including direct participation from patients. The researchers obtained ethical approval from the appropriate institutional review board, which granted them permission to conduct research without obtaining informed consent because of the study’s retrospective design. This research was approved by the Ethics Committee of Xixian Hospital of Shaanxi Provincial People’s Hospital, approval number: 2021HL-23.

## 3 Results

### 3.1 Baseline demographic and clinical characteristics

**FIGURE 1:**
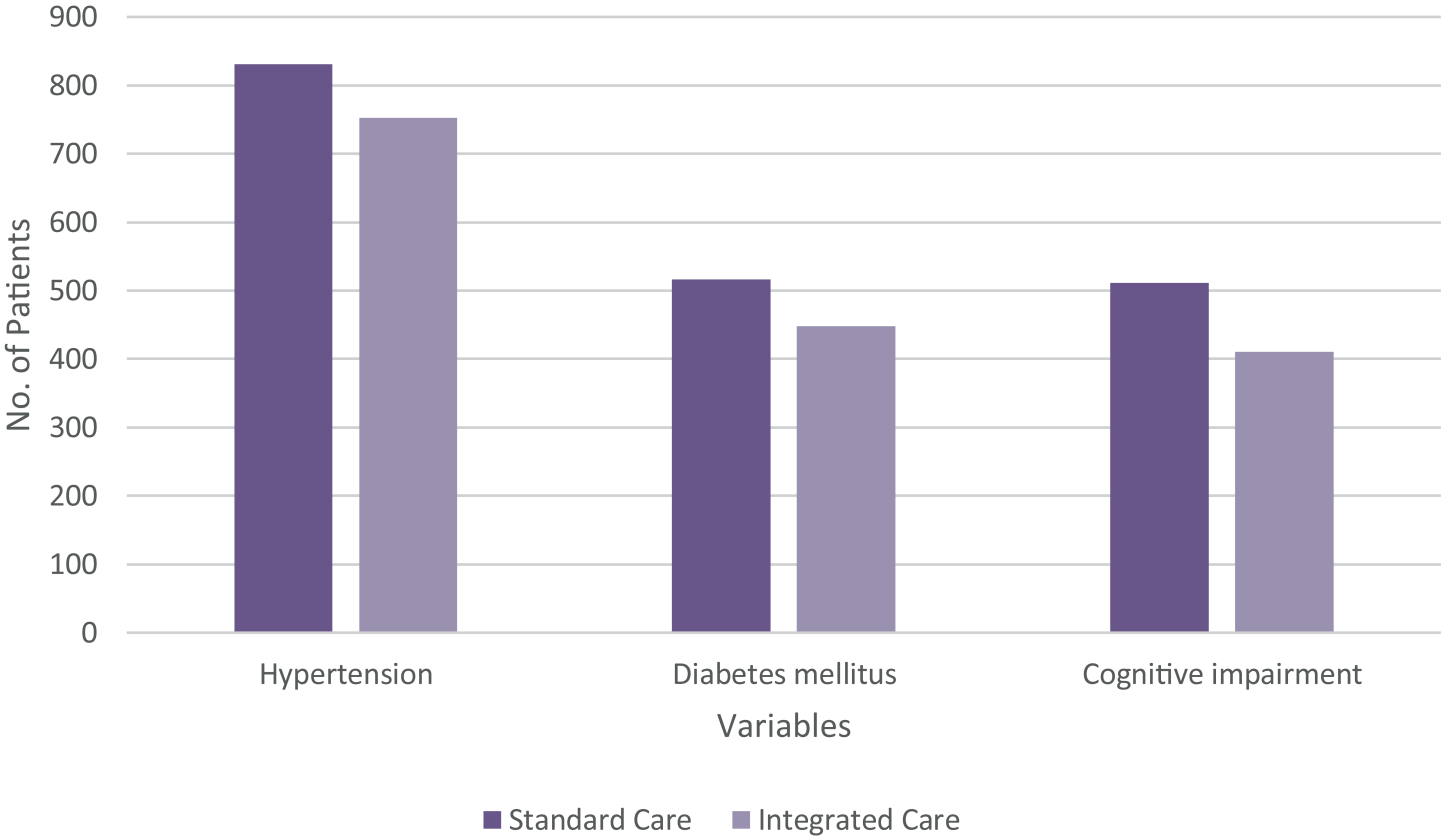
Clinical characteristics.

The baseline characteristics were generally comparable between the patients in the standard and integrated care models (standard care: *n* = 1,190, integrated care: *n* = 1,110). There was no difference between the two groups in terms of mean age (75.6 ± 7.3 versus 76.1 ± 7.1 years, respectively; *p* = 0.08) and gender composition. However, the patients enrolled in the standard care program showed greater rates of social isolation (37.9% vs. 33.5%, *p* = 0.03), multimorbidity (70.8% vs. 66.0%, *p* = 0.02), cognitive impairment (42.9% vs. 36.9%, *p* = 0.01), frailty (34.8% vs. 29.0%, *p* = 0.004), and polypharmacy (51.4% vs. 46.3%, *p* = 0.01) (Figure 1 and Table 1).

**TABLE 1:** Baseline demographic and clinical characteristics by care model.

Variable	Total (*n* = 2,300)	Standard Care (*n* = 1,190)	Integrated care (*n* = 1,110)	*P*-value	SMD
Age (years)	75.8 ± 7.2	75.6 ± 7.3	76.1 ± 7.1	0.08	0.07
Female	1,276 (55.5%)	642 (54.0%)	634 (57.1%)	0.14	0.06
Education ≥ 12 yrs	918 (39.9%)	456 (38.3%)	462 (41.6%)	0.12	0.07
Living alone	823 (35.8%)	451 (37.9%)	372 (33.5%)	0.03	0.09
BMI ≥ 30 kg/m^2^	644 (28.0%)	349 (29.3%)	295 (26.6%)	0.18	0.06
Multimorbidity ( ≥ 2)	1,575 (68.5%)	842 (70.8%)	733 (66.0%)	0.02	0.10
Frailty (FI ≥ 0.25)	736 (32.0%)	414 (34.8%)	322 (29.0%)	0.004	0.13
Depression (GDS ≥ 10)	748 (32.5%)	401 (33.7%)	347 (31.3%)	0.21	0.05
Polypharmacy ( ≥ 5 meds)	1,126 (49.0%)	612 (51.4%)	514 (46.3%)	0.01	0.10
Charlson comorbidity index	3.1 ± 1.4	3.2 ± 1.4	3.0 ± 1.3	0.06	0.07

### 3.2 Relevance and severity of geriatric syndromes

**FIGURE 2:**
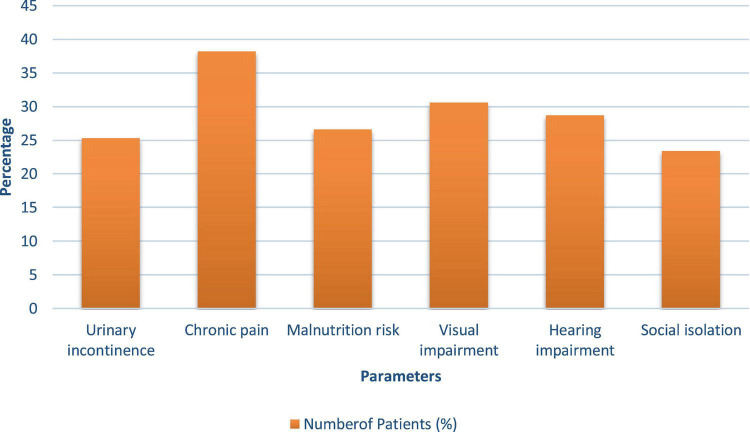
Severity of geriatric syndromes.

The presence of geriatric syndromes is clearly depicted in Table 2. The prevalence rate for cognitive impairment was 40.0% (MoCA 22.4 ± 2.6), whereas 17.7% suffered from dementia. The prevalence of frailty in the study subjects was 32.0% (FI 0.31 ± 0.05). On the other hand, depressive symptoms were present in 32.5% of the sample population (GDS 9.8 ± 3.1). Falls within a year accounted for 44.3% of cases, while chronic pain was experienced by 38.2% of patients (VAS 5.2 ± 1.9). The prevalence rate of malnutrition risk in the study population was 26.6% (MNA-SF 9.6 ± 2.1). Social isolation was found in 23.4% of patients, while visual and auditory impairments were experienced by 30.6 and 28.7% of the study population, respectively. Furthermore, polypharmacy occurred in 49.0%, averaging 6.4 drugs per subject (Figure 2 and Table 2).

**TABLE 2:** Prevalence and severity of geriatric syndromes (baseline, retrospective assessment).

Condition	*n* (%)	95% CI	Severity score	Scale
Cognitive impairment	921 (40.0%)	38.0–42.0	22.4 ± 2.6	MoCA (0–30)
Dementia (diagnosis)	407 (17.7%)	16.1–19.3	–	EHR diagnosis
Frailty	736 (32.0%)	30.1–33.9	0.31 ± 0.05	FI (0–1)
Depression	748 (32.5%)	30.6–34.4	9.8 ± 3.1	GDS (0–15)
Falls (past year)	1,018 (44.3%)	42.3–46.3	1.6 ± 0.8	EHR documentation
Polypharmacy	1,126 (49.0%)	47.0–51.0	6.4 ± 1.8 meds	Prescription data

### 3.3 Health service utilization

Among the elderly patients during the 24 months of the follow-up period, there was an increased use of healthcare services. The number of hospitalizations was 92 per 100 person-years with a median number of 1 per person (IQR 1–3). Similarly, the number of emergency room visits was high with a median number of 2 per individual and a rate of 134 per 100 person-years. The most common healthcare service used by these patients was outpatient visits with a mean number of 9.2 ± 5.4 and a rate of 460 per 100 person-years. However, the use of skilled nursing facility days and ICU admissions were relatively low with high zero-inflation (Table 3).

**TABLE 3:** Health service utilization (24-month follow-up).

Utilization measure	Median (IQR)	Mean ± SD	Rate/100 PY	Zero-inflation
Hospital admissions	1 (1–3)	1.8 ± 1.3	92	12%
Emergency visits	2 (1–4)	2.6 ± 1.9	134	8%
Outpatient visits	8 (5–12)	9.2 ± 5.4	460	4%
Skilled nursing stays	0 (0–2)	0.8 ± 1.2	41	55%
Home-health episodes	2 (1–3)	2.1 ± 1.5	105	38%
ICU admissions	0 (0–1)	0.3 ± 0.6	15	78%

### 3.4 Functional and quality-of-life changes over 24 months

**FIGURE 3:**
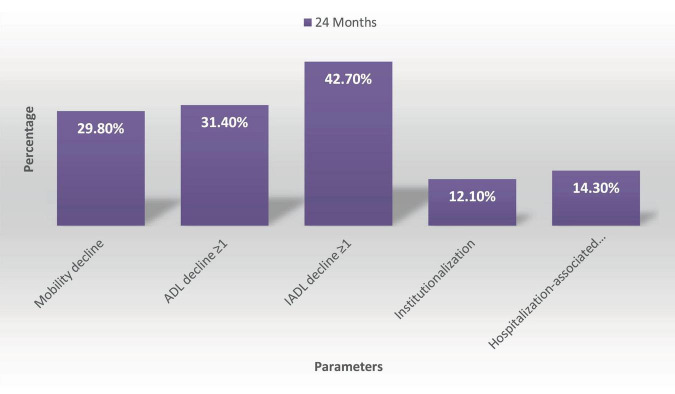
Quality-of-life changes.

In the course of 2 years of follow-up, functional ability and quality of life significantly deteriorated. ADL index score dropped from 4.9 ± 1.1 to 4.4 ± 1.3 (mean difference: −0.5, 95% confidence interval [CI]: −0.6 to −0.4; *p* < 0.001), whereas IADL index score dropped from 5.6 ± 1.8 to 4.9 ± 2.1 (mean difference: −0.7, 95% CI: −0.9 to −0.5; *p* < 0.001). Decline in quality of life was observed through decrease in EQ-5D scores, which went down from 0.68 ± 0.17 to 0.64 ± 0.19 (*p* < 0.01). The incidence of clinical worsening of ADL and IADL was reported at 31.4 and 42.7%, respectively. Institutionalization and hospital-related disability occurred in 12.1 and 14.3% of patients, respectively (Figure 3 and Table 4).

**TABLE 4:** Functional and quality-of-life changes (retrospective 24-month change).

Measure	Baseline	24 Months	Mean change (95% CI)	*P*-value	Effect size
ADL (0–6)	4.9 ± 1.1	4.4 ± 1.3	−0.5 (−0.6 to −0.4)	< 0.001	0.41
IADL (0–8)	5.6 ± 1.8	4.9 ± 2.1	−0.7 (−0.9 to −0.5)	< 0.001	0.38
EQ-5D	0.68 ± 0.17	0.64 ± 0.19	−0.04 (−0.06 to −0.02)	< 0.01	0.24
Self-rated health	3.1 ± 0.9	3.3 ± 1.2	+0.2 (0.1–0.3)	< 0.05	0.18

### 3.5 Multivariable negative binomial model—hospital admissions

This study found that there was a significantly lower rate of admissions among those receiving integrated care than those receiving conventional care (adjusted IRR = 0.78, 95% CI 0.71–0.87, *p* < 0.001), representing a 22% reduction in admission rates after controlling for possible confounders. Advanced age, multimorbidity, frailty, cognitive decline, depression, polypharmacy, history of falling, and living alone predicted admission (all *p* < 0.05), but education and obesity did not significantly predict admission rates. It appears from this study that multidisciplinary integrated care can decrease unnecessary admissions, and vulnerability and multimorbidity in the elderly predict healthcare utilization (Table 5).

**TABLE 5:** Multivariable negative binomial model—hospital admissions.

Predictor	Adjusted IRR	95% CI	*P*-value
Integrated care	0.78	0.71–0.87	<0.001
Age (per year)	1.02	1.01–1.03	0.004
Multimorbidity	1.19	1.10–1.29	<0.001
Frailty	1.41	1.28–1.56	<0.001
Cognitive impairment	1.29	1.17–1.42	<0.001
Depression	1.12	1.03–1.22	0.02
Living alone	1.10	1.01–1.21	0.04
Polypharmacy	1.16	1.07–1.27	0.001
Prior falls	1.18	1.08–1.30	0.002
Education ≥ 12 years	0.92	0.84–1.01	0.08
BMI ≥ 30	1.04	0.94–1.15	0.44

### 3.6 Cognitive outcomes over 24 months

There was a significant advantage for cognitive function among the patients receiving integrated care relative to conventional care over the 24-month follow-up period. Both study groups witnessed a decline in MoCA scores, though the decline was less pronounced among the patients receiving integrated care (−1.9 vs. −2.8 points), which represented an adjusted mean difference of 0.86 points (95% CI 0.42–1.30; *p* < 0.001). The conversion rate to dementia was lower in the case of integrated care (6.7% vs. 9.1%; HR 0.71, 95% CI 0.54–0.94; *p* = 0.02). Likewise, the risk of cognitive decline of at least 2 points was significantly reduced for patients receiving integrated care (34.7% vs. 43.5%; RR 0.80, 95% CI 0.69–0.93; *p* = 0.001) (Table 6).

**TABLE 6:** Cognitive outcomes (24-month change).

Outcome	Standard	Integrated	Adjusted effect	*P*-value
MoCA change	−2.8	−1.9	+0.86 (0.42–1.30)	< 0.001
Dementia conversion	9.1%	6.7%	HR 0.71 (0.54–0.94)	0.02
Cognitive decline ≥ 2 pts	43.5%	34.7%	RR 0.80 (0.69–0.93)	0.001

### 3.7 Functional decline by geriatric syndrome burden

Greater numbers of geriatric syndromes were related to functional decline and increased health-care usage. Those people who had 2–3 syndromes demonstrated more ADL decline (29.4%; adjusted OR = 1.72, 95% CI 1.38–2.14) and IADL decline (38.8%; OR 1.63, 95% CI 1.32–2.02) as well as had a greater hospitalization rate (IRR = 1.29, 95% CI 1.14–1.45, which corresponds to a 47.8% increase). People who suffered from 4+ geriatric syndromes had significantly increased rates of ADL decline (46.0%; OR = 2.91, 95% CI 2.32–3.65), IADL decline (55.1%; OR 2.48, 95% CI 2.01–3.06), and greater hospitalization rate (59.3%; IRR = 1.61, 95% CI 1.43–1.81). Thus, the relationship between geriatric syndrome burden and both health-care use and functional decline proved to be dose-related (Table 7).

**TABLE 7:** Functional decline by geriatric syndrome burden.

Syndrome count	n	ADL decline ≥ 1	Adjusted OR (95% CI)	IADL decline ≥ 1	Adjusted OR (95% CI)	Hospitalization	Adjusted IRR (95% CI)
0–1	498	18.5%	Reference	25.7%	Reference	36.9%	Reference
2–3	962	29.4%	1.72 (1.38–2.14)	38.8%	1.63 (1.32–2.02)	47.8%	1.29 (1.14–1.45)
≥ 4	840	46.0%	2.91 (2.32–3.65)	55.1%	2.48 (2.01–3.06)	59.3%	1.61 (1.43–1.81)

### 3.8 Propensity score–matched characteristics

In regard to propensity score matching, the standard and integrated care groups had excellent balance at baseline as evidenced by standardized mean differences (SMDs) < 0.05 on all the variables examined. Both the age means and standard deviations were comparable between the two groups (75.9 ± 7.1 vs. 76.0 ± 7.2 years; SMD = 0.02) as well as the percentage of females in both samples (56.7% vs. 56.0%; SMD = 0.01). Multimorbidity rates (67.4% vs. 66.6%), frailty (31.4% vs. 30.5%), cognitive impairments (38.8% vs. 38.2%), and polypharmacy (48.9% vs. 47.5%) were also similar between the groups (Table 8).

**TABLE 8:** Propensity score–matched characteristics (1:1 retrospective matching).

Variable	Standard (*n* = 980)	Integrated (*n* = 980)	SMD
Age	75.9 ± 7.1	76.0 ± 7.2	0.02
Female	56.7%	56.0%	0.01
Multimorbidity	67.4%	66.6%	0.02
Frailty	31.4%	30.5%	0.02
Cognitive impairment	38.8%	38.2%	0.01
Polypharmacy	48.9%	47.5%	0.03

### 3.9 Post-match utilization and outcomes

**FIGURE 4:**
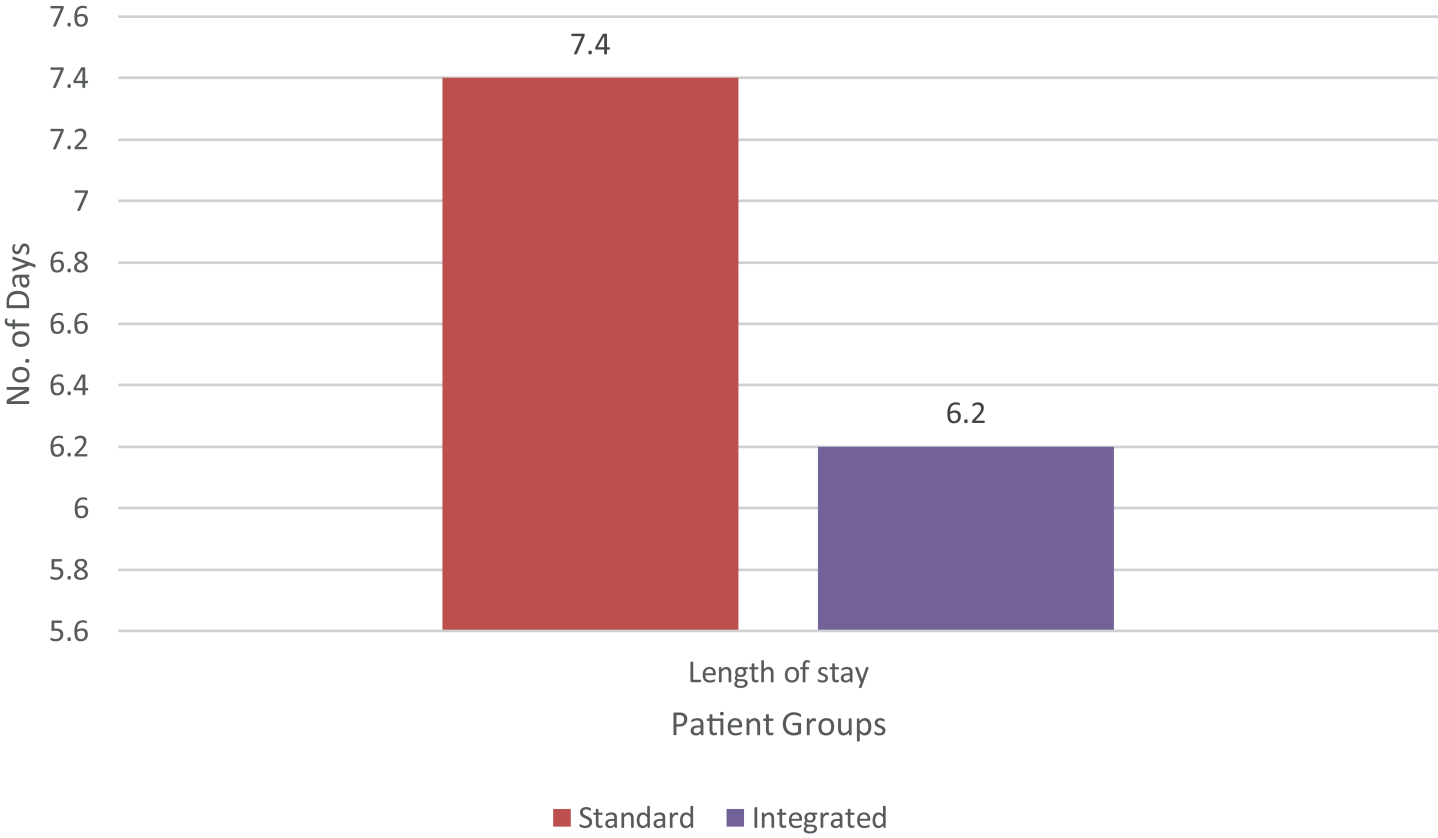
Length of hospital stay.

Following matching, patients in the integrated care program utilized less healthcare services than those in the standard care program. First, the number of patients who had been hospitalized was lower (38.2% vs. 46.8%; IRR 0.82, 95% CI 0.73–0.92; *p* = 0.001). Second, 30-day readmissions rates were significantly reduced (17.9% vs. 22.1%; OR 0.81, 95% CI 0.69–0.95; *p* = 0.01), showing that their post-hospitalization care was more successful. Moreover, the frequency of ICU admissions was lowered (7.1% vs. 9.8%; OR 0.73, 95% CI 0.57–0.93; *p* = 0.01), and the mean length of their stay in the hospital was shortened (6.2 vs. 7.4 days; *p* = 0.02) (Figure 4 and Table 9).

**TABLE 9:** Post-match utilization and outcomes.

Outcome	Standard	Integrated	Adjusted OR/IRR	95% CI	*P*-value
≥ 1 Admission	46.8%	38.2%	IRR 0.82	0.73–0.92	0.001
30-Day readmission	22.1%	17.9%	OR 0.81	0.69–0.95	0.01
ICU admission	9.8%	7.1%	OR 0.73	0.57–0.93	0.01
SNF utilization	28.3%	23.0%	OR 0.81	0.69–0.94	0.006

### 3.10 Mortality over 24-month follow-up

Within the 24-month follow-up period, survival was greater among participants in the integrated care program than those receiving standard care. Participants who received integrated care had fewer deaths than their counterparts who received standard care (10.8% vs. 14.2%). The proportional hazard assumption was supported by data from Schoenfeld test with a probability value of 0.42, implying model validity. Though lower rates of death due to cardiovascular issues and infections were found among patients receiving integrated care (4.9% vs. 6.4% and 2.1% vs. 3.2%), there was no statistical significance between the two groups regarding both causes of death (CV mortality hazard ratio 0.77, *p* = 0.13; infection mortality hazard ratio 0.66, *p* = 0.10) (Table 10).

**TABLE 10:** Mortality (24-month follow-up).

Outcome	Standard	Integrated	HR	95% CI	*P*-value
All-cause mortality	14.2%	10.8%	0.76	0.62–0.93	0.008
CV mortality	6.4%	4.9%	0.77	0.55–1.08	0.13
Infection death	3.2%	2.1%	0.66	0.40–1.09	0.10

### 3.11 Subgroup analysis

The results of exploratory subgroup analyses suggested that integrated care was most effective in reducing mortality in frail patients (HR = 0.72, 95% CI 0.63–0.84; p-interaction = 0.03), with a relatively larger reduction of mortality risk in this high-risk patient population. For multimorbid patients (at least three medical problems), there was a favorable trend without reaching statistical significance (HR = 0.77, 95% CI 0.66–0.89; p-interaction = 0.18), which means no presence of effect modification in this subgroup. Moreover, there was a tendency for lower hazard ratios to be found for individuals with cognitive disorders (HR = 0.79) and depression (HR = 0.83), though they did not reach statistical significance (p-interaction = 0.09; p-interaction = 0.08) (Table 11).

**TABLE 11:** Subgroup analysis.

Subgroup	HR	95% CI	p-interaction
Frail	0.72	0.63–0.84	0.03
Multimorbidity ≥ 3	0.77	0.66–0.89	0.18
Cognitive impairment	0.79	0.67–0.94	0.09
Depression	0.83	0.71–0.98	0.08

### 3.12 Mediation analysis

The counterfactual mediation analysis demonstrated the existence of a significant total effect of the intervention on outcomes [estimate = -0.24, 95% confidence interval (CI): −0.30 to −0.18; *p* < 0.001]. This effect was still significant when taking into account the mediating factors (−0.18, 95% CI: −0.25 to −0.11; *p* < 0.01), meaning that the benefits could still be observed when other explanatory pathways were considered. The reduction in frailty explained the mediating role of frailty between the intervention and the outcome, having a significant indirect effect of −0.06 (95% CI: −0.11 to −0.02; *p* = 0.02). This means that part of the positive benefits was due to reducing frailty, whereas the remaining part was explained by other mechanisms (Table 12).

**TABLE 12:** Mediation analysis.

Effect	Estimate	95% CI	*P*-value
Total effect	−0.24	−0.30 to −0.18	<0.001
Direct effect	−0.18	−0.25 to −0.11	<0.01
Indirect (frailty)	−0.06	−0.11 to −0.02	0.02

### 3.13 Sensitivity analyses

**FIGURE 5:**
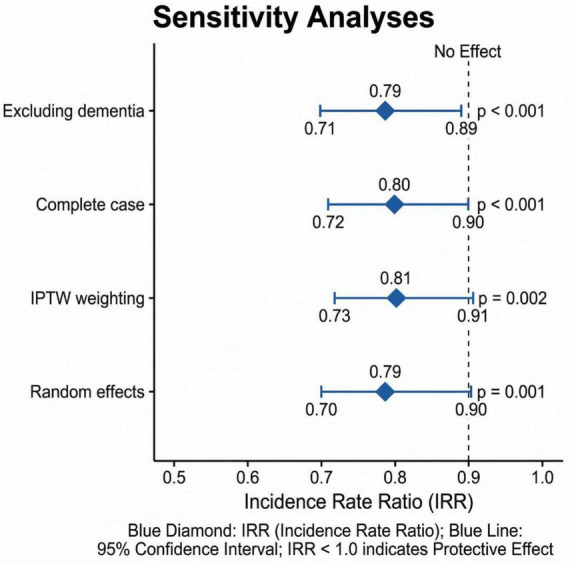
Forest plot of sensitivity analyses.

All sensitivity analyses indicated consistent results supporting the main findings, confirming that an association between integrated care and lower odds of being hospitalized persisted irrespective of the methods used. The IRR after removing individuals with dementia was still statistically significant (IRR 0.79, 95% CI 0.71–0.89; *p* < 0.001). Complete-case analysis also confirmed the result (IRR 0.80, 95% CI 0.72–0.90; *p* < 0.001), suggesting little effect of missing values on the result. Inverse probability of treatment weighting provided further evidence of protection (IRR 0.81, 95% CI 0.73–0.91; *p* = 0.002), and the random-effect model also generated similar figures (IRR 0.79, 95% CI 0.70–0.90; *p* = 0.001) (Figure 5).

### 3.14 Predictors of functional decline

**FIGURE 6:**
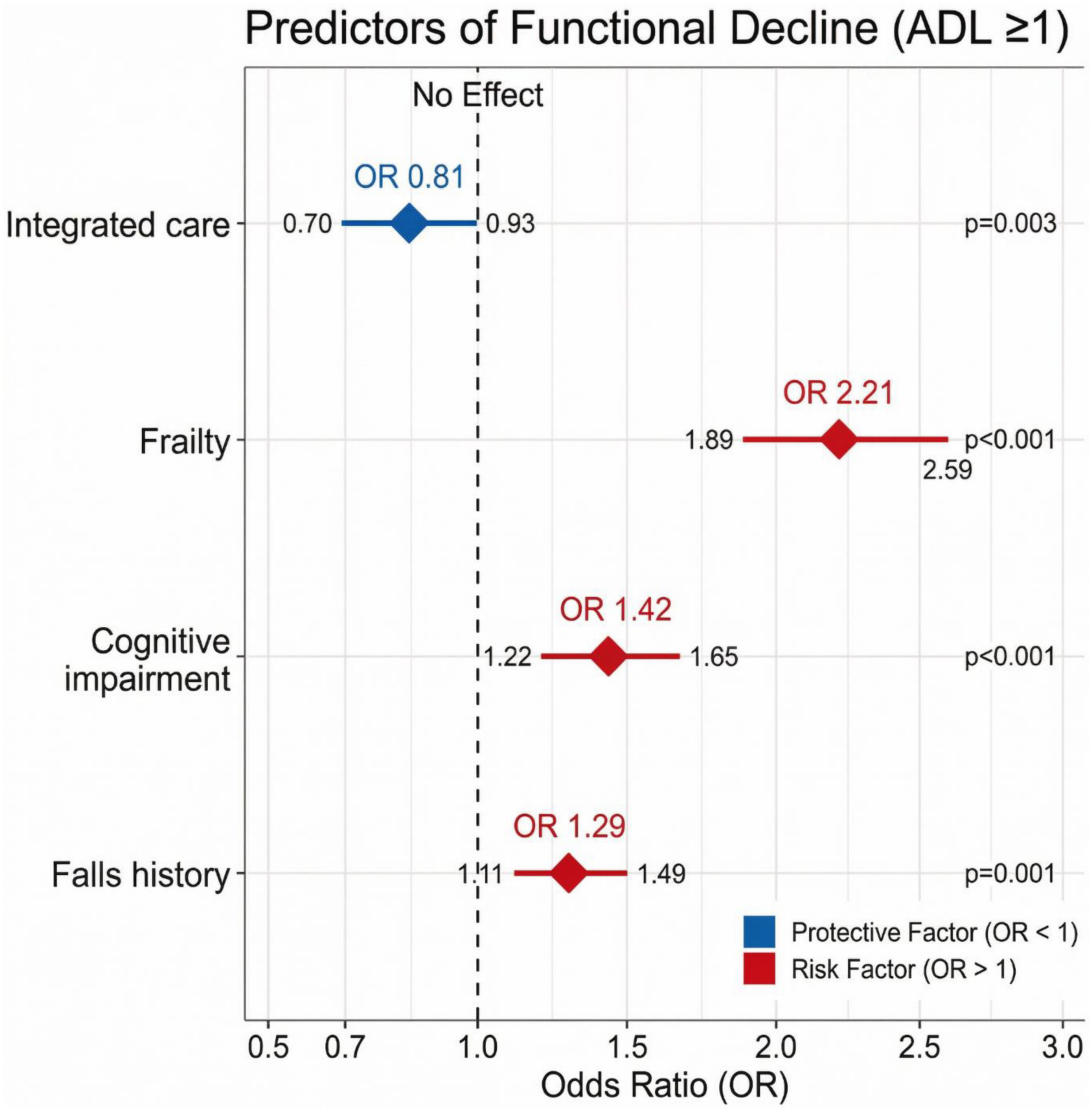
Forest plot of the predictors of functional decline.

Determinants of physical functioning decline were explored in this study. The predictive model for functional decline (loss of at least one ADL) showed good discrimination ability (AUC 0.78) and decent calibration (H-L test *p* = 0.31). Integrated care had a negative correlation with functional decline and suggested a protective impact on functional autonomy. Being a frail patient was found to be the most significant determinant (OR 2.21, 95% CI 1.89–2.59; *p* < 0.001) while being cognitively impaired (OR 1.42, 95% CI 1.22–1.65; *p* < 0.001) and having a history of falls (OR 1.29, 95% CI 1.11–1.49; *p* = 0.001) increased the odds of functional decline as well (Figure 6).

### 3.15 Correlation matrix—spearman

**FIGURE 7:**
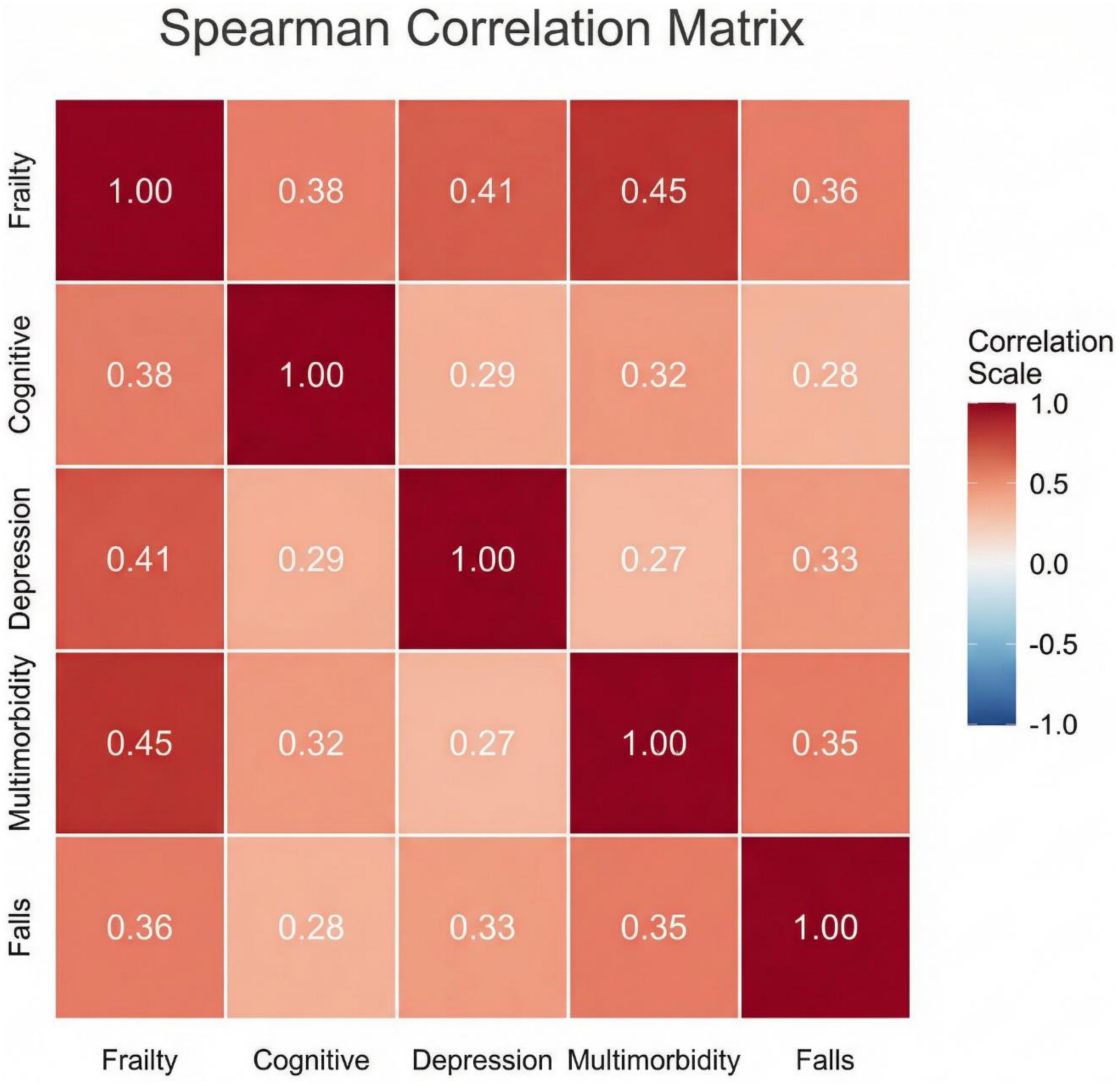
Heatmap.

Functional decline (ADL loss ≥ 1) occurred and had a high ability to discriminate (AUC 0.78) and proper calibration (Hosmer–Lemeshow test *p*-value 0.31). The integrated care strategy correlated negatively with the risk of functional deterioration, implying its role as a protective factor against losing independence. The most significant predictor for functional decline was frailty (OR 2.21, 95% CI 1.89–2.59; *p* < 0.001). Other risk factors included cognitive impairments (OR 1.42, 95% CI 1.22–1.65; *p* < 0.001) and falls (OR 1.29, 95% CI 1.11–1.49; *p* = 0.001). In conclusion, geriatric syndromes played an essential part in functional decline (Figure 7).

### 3.16 Model diagnostics

Model diagnostics suggested that overall model performance was satisfactory irrespective of the analytic approach employed. The principal negative binomial regression model yielded a goodness-of-fit measure of 4,123 AIC, a pseudo *R*^2^ of 0.18, and a dispersion coefficient of 1.74, thus reflecting minor over-dispersion but suitability for count data regression. Following matching, there was improved performance in model diagnostics, whereby the AIC value reduced to 3,987, the pseudo *R*^2^ increased to 0.21, and the dispersion value dropped to 1.31. The Cox proportional hazard regression for mortality performed well in terms of discrimination (C-index of 0.73) and met the assumption of proportional hazard ratios. Similarly, the logistic regression for ADL deterioration had excellent discrimination (C-index 0.78), calibration, and a pseudo *R*^2^ of 0.24 (Table 13).

**TABLE 13:** Model diagnostics.

Model	AIC	Pseudo *R*^2^	Dispersion	C-index	Calibration
NB (primary)	4,123	0.18	1.74	–	Adequate
NB (matched)	3,987	0.21	1.31	–	Improved
Cox (mortality)	–	–	–	0.73	Good
Logistic (ADL)	2,811	0.24	–	0.78	Good

## 4 Discussion

The findings from the retrospective observational study showed that the integrated multi-disciplinary approach had been linked with reduced hospital admissions, minimized functional decline, positive effects on cognitive functions, and reduced all-cause mortality among patients suffering from geriatric syndromes in combination with multimorbidity (19–23). Therefore, this finding adds to the existing body of knowledge suggesting that coordinated care of persons at increased risk of being vulnerable may improve health and decrease healthcare usage (15, 24, 25).

As for the baseline characteristics, patients in the study cohort were found to have experienced a number of geriatric syndromes, including cognitive impairment, frailty, depression, multimorbidity, and polypharmacy. These results are similar to those obtained in previous epidemiological studies indicating that geriatric syndromes are common conditions which often appear at once and lead to disability, hospitalization, and even mortality among the older population groups (26–31).

One of the most significant results obtained during the implementation of the program was the 22% decrease in hospitalizations due to integrated care. The same result was found in a number of studies on CGA, comprehensive management of chronic diseases, and transitional care initiatives (32–34). Moreover, the patients enrolled in the integrated care program showed a decrease in their chances for 30-day re-hospitalization, admission to ICUs, and the need for skilled nursing facilities (35, 36). This might be caused by the coordinated actions of different specialists involved in the program.

Maintenance of functionality became one more important achievement of the program. Patients included in the study showed lower chances of functional decline, which was estimated according to the decrease in the level of performance of IADLs and ADLs over the 24-month follow-up period (37–40). The presence of frailty became the primary reason for the loss of functions; therefore, the development of programs to prevent frailty, rehabilitate elderly people, optimize pharmacotherapy, and follow up should be continued (41–46).

The integrated care approach was also linked to positive cognition, where the decline in MoCA scores and the rate of conversion to dementia were less pronounced. Other longitudinal and multidomain intervention studies have also found that the management of risk factors for cardiovascular diseases, depression, frailty, and social isolation can reduce cognitive decline (47–49). Reduced rates of cognitive decline in the integrated care group could be due to the combined impact of repeated monitoring of cognition, drug regimen evaluation, social and psychological care, and chronic disease management.

Mortality analysis showed lower all-cause mortality in participants enrolled in integrated care. It seems that frailty partially mediated the relationship between integrated care and mortality. This is consistent with previous research demonstrating that frailty mediates the biological connection between multimorbidity and negative outcomes (50–55). The subgroup analysis indicated that frailty patients may gain the most benefit from integrated geriatric care.

Moreover, there was shown a dose-response association between the burden of geriatric syndromes and the occurrence of negative outcomes. People who had several syndromes at once tended to have much more frequent hospitalizations and a greater chance of developing functional decline. As expected, polypharmacy proved to be another strong determinant of negative consequences, consistent with previous studies that established links between high medication burden and hospitalization and functional impairments (56–61).

It is necessary to recognize several advantages in relation to methodology used by the authors. This study relied on a large real-world sample size, detailed clinical information, longitudinal data, multivariable analyses, sensitivity analysis, and propensity score matching to control for the impact of measured confounders (62–64).

However, there are some limitations worth noting as well. For instance, the observational design prevents drawing definitive causal conclusions, while propensity score matching may not entirely exclude confounding variables. Additionally, randomization was not applied for group assignment, meaning some unknown factors, including socioeconomic status, caregiver support, and access to medical care, might have influenced results. Moreover, electronic health records used by the authors could potentially be prone to the issue of ascertainment bias. The study was conducted at a single center only and may thus lack external validity (65, 66).

There are a number of limitations in this research that must be taken into account for proper interpretation of its results. The use of a retrospective observational design does not allow drawing causal conclusions regarding the linkages between integrated care and the outcomes observed in the study sample. Despite application of sophisticated analytical techniques, such as propensity score matching and multivariate adjustment, residual and unmeasured confounding could not be completely excluded. Both the exposure variable and some outcome variables were defined through data coded in EHRs, and thus they were prone to classification errors.

Certain covariates were obtained from routine clinical documentation, hence the probability of measurement variability and incomplete ascertainment remains valid. The issue of missing data was addressed properly using analytical techniques, yet some level of residual bias could be present. Fourth, because the study sample was comprised of those individuals who had available longitudinal data for analysis, there might be selection bias involved. The study was carried out in a single healthcare setting and among a relatively homogeneous group of patients, limiting its external validity.

## 5 Conclusion

From the retrospective observational analysis in this case, it is clear that there is an association between integrated geriatric care, which is multidisciplinary, and the reduction in admission rates to hospitals as well as the utilization of medical care, improvement in terms of cognitive function and physical ability as well as overall low death rates in patients with multiple morbidities and geriatric syndromes. From this research work, frailty, cognitive problems, polypharmacies and multimorbidity were found out to have a direct association with bad health outcomes. There might possibly be some ways through which coordinated geriatric care pathways such as medication optimization, rehabilitation programs and multidisciplinary geriatric assessments can help maintain good health outcomes for high-risk elderly patients. Though causality cannot be drawn since this is a retrospective study, the consistency in results from the different analyses gives credibility to the results. It calls for further prospective multicenter studies and randomized controlled trials to verify the effectiveness of integrated care programs.

## Data Availability

The raw data supporting the conclusions of this article will be made available by the authors, without undue reservation.
